# Monocyte programmed death ligand-1 expression after 3–4 days of sepsis is associated with risk stratification and mortality in septic patients: a prospective cohort study

**DOI:** 10.1186/s13054-016-1301-x

**Published:** 2016-05-09

**Authors:** Rui Shao, Yingying Fang, Han Yu, Lianxing Zhao, Zhifeng Jiang, Chun-Sheng Li

**Affiliations:** Department of Emergency Medicine, Beijing Chao-yang Hospital, Capitcal Medical University, 8# Worker’s Stadium South Road, Chao-yang District, Beijing, 100020 China

**Keywords:** PD-L1, Immunosuppression, Septic shock, Mortality

## Abstract

**Background:**

Septic shock is a major healthcare problem with a high mortality rate that might be caused by immunosuppression. Programmed cell death receptor-1 (PD-1) and programmed cell death receptor ligand-1 (PD-L1), which are co-inhibitory receptor molecules, participate in sepsis-induced immunosuppression. In this study, we investigated which PD-1-related molecules can be used to evaluate the risk stratification and prognosis of septic patients. Furthermore, we explored the prognostic significance of a combination of ideal predictors and conventional clinical risk parameters in septic shock patients.

**Methods:**

In total, 29 healthy controls, 59 septic patients, and 76 septic shock patients were enrolled in this study. Considering that the focus of the research was on the second phase of sepsis, blood samples were obtained at days 3–4 after the onset of systemic inflammatory response syndrome (SIRS). PD-1 and PD-L1 expression were measured on circulating CD4^+^ T cells, CD8^+^ T cells, and monocytes (PD-L1 only) by flow cytometry.

**Results:**

Our results showed that only monocyte PD-L1 expression gradually increased, based on the increasing severity of disease (*P* < 0.001). Similarly, multivariate logistic regression analysis showed that only monocyte PD-L1 expression was an independent predictor of 28-day mortality in septic shock patients. Area under the receiver operating characteristic curve analysis of the combination of monocyte PD-L1 expression and conventional clinical risk parameters indicated a more significant prognostic ability than analysis of each parameter alone.

**Conclusion:**

Our study demonstrated that, among PD-1-related molecules, only monocyte PD-L1 expression after 3–4 days of sepsis was associated with risk stratification and mortality in septic patients. Furthermore, measurement of monocyte PD-L1 expression was a promising independent prognostic marker for septic shock patients.

## Background

Septic shock is a major healthcare problem with a high mortality rate [1]. Early and aggressive support treatment has not improved survival in patients with septic shock [2]. Postmortem studies of septic patients highlighted that immunosuppression may be a key cause of increased mortality [3, 4]. Indeed, the majority of nonsurviving patients with septic shock died in the stage of immunosuppression [5]. The immunosuppression phase is characterized by increased lymphocyte apoptosis, increased numbers of regulatory T cells, increased suppression of cytokine production, and decreased human leukocyte antigen-DR (HLA-DR) expression [6–9]. Programmed cell death receptor-1 (PD-1) and programmed cell death receptor ligand-1 (PD-L1), which are co-inhibitory receptor molecules, play major roles in sepsis-induced immunosuppression [4].

PD-1 is expressed on activated T cells, natural killer cells, and B cells [10]. Its ligand, PD-L1, is broadly expressed on hematopoietic and nonhematopoietic cells [11]. The PD-1/PD-L1 pathway exerts inhibitory effects by regulating T-cell activation, tolerance, and immunopathology [12, 13]. Although many studies have explored the roles of the PD-1/PD-L1 pathway in septic animals and patients, few studies have explored the relationship between PD-1-related molecules and the risk stratification of septic patients, and evaluated which PD-1-related molecules are useful biomarkers to predict mortality during the immunosuppressive phase of septic shock. In addition, most previous reports of PD-1-related molecules during sepsis focused on single measurements of immunosuppression. The correlation of PD-1-related molecules with conventional clinical risk parameters may be more useful for predicting 28-day mortality. In this study, we evaluated whether PD-1 and PD-L1 expression on circulating CD4^+^ T cells, CD8^+^ T cells, and monocytes (PD-L1 only) at days 3–4 of the onset of sepsis can be used to evaluate the risk stratification and prognosis of septic patients. Furthermore, we explored the prognostic significance of a combination of ideal predictors and conventional clinical risk parameters in septic shock patients.

## Methods

### Patients

The patients in this study were from the emergency department (ED) of Beijing Chao-yang Hospital. There are about 250,000 ED admissions per year in this university teaching hospital. Patients admitted to the ED at days 1–2 after the onset of signs of systemic inflammatory response syndrome (SIRS) were evaluated for possible enrollment according to the inclusion and exclusion criteria. The eligible patients were treated according to the international guidelines for management of septic shock [2]. The beginning of vasopressive therapy was defined as the onset of the septic shock. Considering the investigation’s focus on the second phase of sepsis, blood samples were obtained at days 3–4 after the onset of SIRS [5, 14].

According to the diagnostic criteria of the 2001 SCCM/ESICM/ACCP/ATS/SIS International Sepsis Definitions Conference [15], sepsis was defined by an identifiable site of infection, which was evidence of a systemic inflammatory response (SIRS) manifested by at least two of the following criteria: (a) body temperature >38.5 °C or <36 °C; (b) heart rate >90 beats per minute; (c) respiratory rate >20 breaths per minute or PaCO_2_ <32 mmHg; and (d) white cell count >12,000/mm^3^ or <4000/mm^3^, or percentage of immature neutrophils >10 %. Septic shock was defined as sepsis-induced hypotension despite adequate fluid resuscitation. Sepsis-induced hypotension was defined as a systolic blood pressure (SBP) <90 mmHg or mean arterial pressure (MAP) <70 mmHg or SBP decrease >40 mmHg or less than two standard deviations below normal for age in the absence of other causes of hypotension. According to the criteria of the International Sepsis Forum Consensus Conference on Definitions of Infection [16], the infection was defined on the basis of clinical features, laboratory findings, and an imaging test. The exclusion criteria were: (a) age less than 18 years; (b) patients with HIV infection or cancer if they were treated with a high dose of corticoids or if they presented with an aplasia (polymorphonuclear neutrophil count of less than 0.5 G/L); (c) patients who died within 2 days of the onset of septic shock; (d) the signs of SIRS occurred more than 3 days prior to admission, or patients were transferred from another hospital; and (e) patients who declined to consent.

### Data collection

Clinical characteristics of patients, including age, gender, past medical history, vital signs, and results of correlative laboratory examinations, were recorded at days 3–4 after the onset of septic shock. Sepsis-related organ failure assessment (SOFA) score and simplified acute physiology score II (SAPS II) were calculated according to related clinical and demographic data. This study was approved by the Beijing Chao-yang Hospital Ethics Committee. Written informed consent was obtained from the patients and volunteers. Consents for patients who were unable to provide consent were provided by first-degree relatives. During follow-up the following data were collected: comorbidities (chronic obstructive pulmonary diseases, congestive heart failure, cerebrovascular disease, diabetes), and the outcome after 28 days (survival or death).

### Flow cytometry

**Fig. 1:**
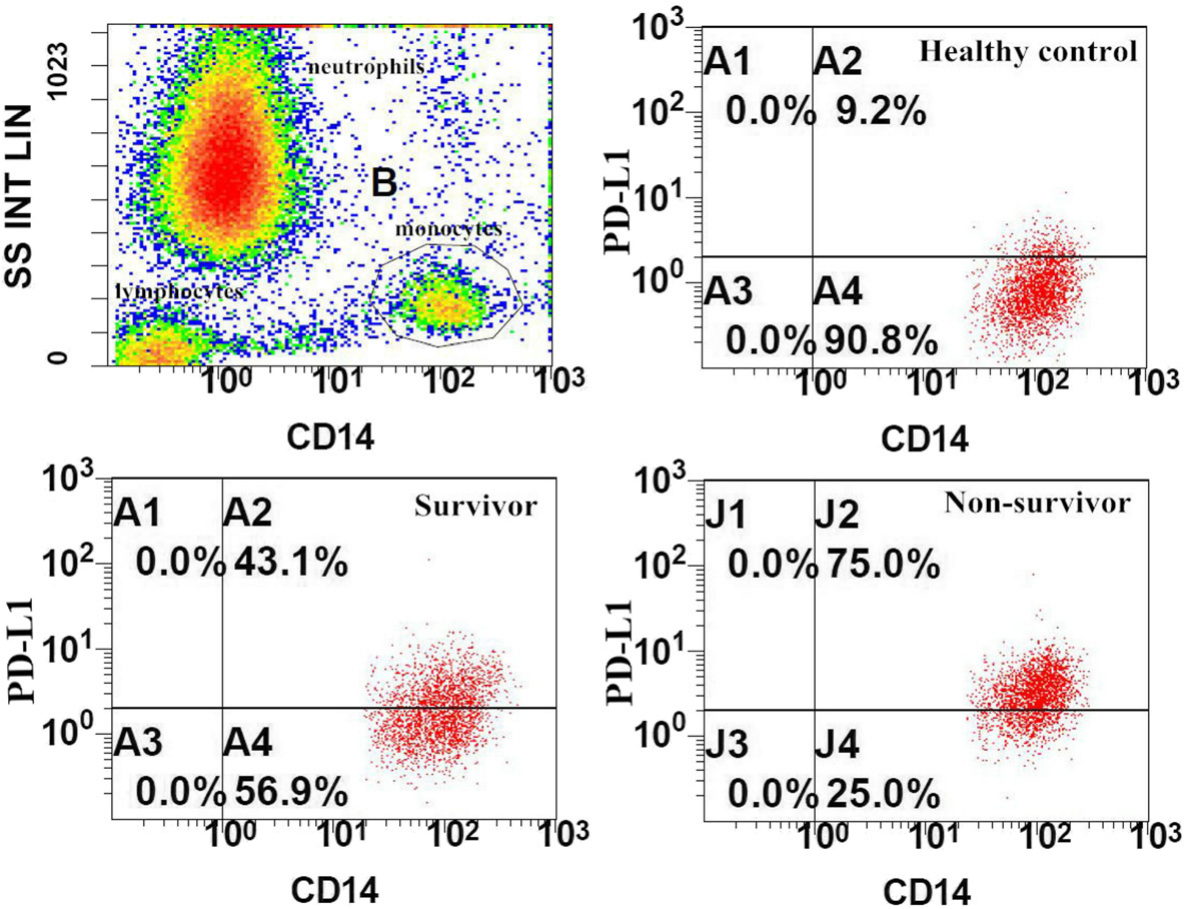
Representative flow dot plots of monocyte gating strategy and the percentage of programmed cell death receptor ligand-1 (*PD-L1*) on monocytes in different groups. The monocytes (gate B) were gated in the dot plots of side-scatter characteristics (*SS*) vs CD14. The multiple dot plots of CD14 vs PD-L1 are representative of the percentage of monocytes in healthy control, survivor, and nonsurvivor groups

Samples of peripheral blood were collected in ethylenediamine tetraacetic acid (EDTA) anticoagulant tubes. The samples were transported to the laboratory at 4 °C within 2 h. Erythrocytes were lysed and cells were stained by a researcher who was blinded to the clinical data. Antibodies were purchased from BD Bioscience (San Jose, CA, USA) or eBioscience (San Diego, CA, USA). According to the manufacturer’s recommendations monoclonal antibodies and their isotype controls were used: BV421-labeled anti-PD1 (5 μl, clone EH12.1), PE-labeled anti-PD-L1 (20 μl, clone M1H1), APC-H7 labeled anti-CD3 (5 μl, clone SK7), FITC-labeled anti-CD4 (5 μl clone OKT4), FITC-labeled anti-CD8 (20 μl, clone RPA-T8), and APC-H7-labeled anti-CD14 (5 μl, clone MφP9) per 100 μl of whole blood. Samples were run on a Gallios™ Flow Cytometer (Beckman Coulter, Inc.) and analyzed using Gallios Software Version 1.0 (Beckman Coulter, Inc.). Lymphocytes were gated by forward scatter (FSC) and side scatter (SSC), and T-cells subsets were further identified by CD3^+^ and CD4^+^ staining. Monocytes were identified by CD14^+^ staining (Fig. 1). At least 3000 cells were analyzed from each sample. The threshold was defined using an isotype control. Results are expressed as percentages and the mean of fluorescence intensities (MFI).

### Statistical analysis

The baseline characteristics were described as frequencies, percentages, median and inter-quartile ranges. Comparisons between groups were made using the Pearson *χ*^2^ test for categorical data and the Mann-Whitney test for continuous variables. For multi-group comparisons, the Kruskal-Wallis test was applied. Age, white blood cell (WBC), lymphocytes, SOFA score, and SAPS II were stratified using cutoff values based on the population median [5]. PD-1 and PD-L1 expressions were stratified using the optimal threshold indicated by the receiver operating characteristic (ROC) curve. Using cutoff values determined by ROC curves, Kaplan-Meier survival curves were established, and the log-rank test was applied for the comparisons of survival distributions. Binary logistic regression was used to identify the variables associated with 28-day mortality in patients with septic shock. Variables with *P* < 0.15 in univariate analysis were conserved in the model. All statistical tests were two-tailed, and *P* < 0.05 was considered statistically significant. All data were analyzed using SPSS 19.0 software (SPSS Inc., Chicago, IL, USA).

## Results

### Patient characteristics

Initially, 29 health controls, 59 septic patients, and 87 septic shock patients were admitted to the ED of Beijing Chao-yang Hospital from June 2014 to September 2015. However, 11 septic shock patients were excluded because they died within 2 days of the onset of septic shock. Thus, 76 septic shock patients were enrolled in this study. Septic patients were divided into the sepsis subgroup and septic shock subgroup according to the disease severity. The demographic and clinical characteristics of the patients are shown in Tables 1 and 2. A comparison of survivors and nonsurvivors showed significant differences in the number of lymphocytes, SOFA score, SAPS II, and comorbidities. Higher SOFA score and SAPS II indicated a high level of severity. The major types of infection were pneumonia (53.9 %) and urinary system infection (USI) (23.7 %).

**Table 1:** Baseline characteristics of the patients

Parameters	Control	Sepsis	Septic shock	*P* value
Number	29	59	76	–
Age (years)	68 (66–75)	71 (66–78)	71 (61–78)	0.497
Male, n (%)	18 (62.1 %)	32 (54.2 %)	37 (48.7 %)	0.458
WBC (×10^9^/L)	6.9 (5.6–7.9)	13.3 (10.9–17.8)	14.9 (11.8–17.2)	<0.001
Lymphocyte (×10^9^/L)	2.8 (2.4–3.1)	1.06 (0.73–1.63)	0.76 (0.54–1.07)	<0.001
SOFA score	0	5 (3–7)	11 (9–14)	<0.001
SAPS II	12 (12–15)	26 (24–32)	53 (46–60)	<0.001
Percentage of PD-1^+^/CD4^+^ T cells (%)	26.2 (23.2–29.9)	34.1 (28.4–44.4)	38.2 (29.2–47.7)	<0.001
MFI of PD-1 on CD4^+^ T cells	6.3 (5.8–6.8)	7.1 (5.2–9.2)	7.5 (6.0–9.4)	0.008
Percentage of PD-1^+^/CD8^+^ T cells (%)	22.6 (18.7–28.2)	31.6 (23.9–46.6)	36.5 (27.3–51.3)	<0.001
MFI of PD-1 on CD8^+^ T cells	5.9 (4.8–6.6)	6.4 (4.2–7.5)	6.6 (4.9–8.4)	0.181
Percentage of PD-L1^+^/CD4^+^ T cells (%)	18.2 (12.3–22.6)	21.5 (16.5–30.5)	21.0 (9.3–30.6)	0.021
MFI of PD-L1 on CD4^+^ T cells	1.8 (1.6–2.2)	1.8 (1.6–2.6)	1.8 (1.5–2.0)	0.230
Percentage of PD-L1^+^/CD8^+^ T cells (%)	22.6 (17.1–26.2)	18.8 (14.9–37.6)	19.5 (10.3–37.4)	0.645
MFI of PD-L1 on CD8^+^ T cells	1.5 (1.4–1.7)	1.6 (1.2–2.1)	1.5 (1.4–1.8)	0.596
Percentage of monocytes expressing PD-L1 (%)	12.9 (10.4–15.3)	29.2 (12.1–43.9)	35.9 (20.4–54.7)	<0.001
MFI of PD-L1 on monocytes	3.4 (3.0–3.9)	4.5 (2.2–7.5)	8.3 (7.7–9.7)	<0.001
Type of infection, n (%)				
Pneumonia	0	30 (50.8 %)	41 (53.9 %)	0.731
IAI	0	7 (11.8 %)	11 (14.5 %)	0.658
CNSI	0	5 (8.5 %)	6 (7.9 %)	0.903
USI	0	17 (28.8 %)	18 (23.7 %)	0.500
Number of comorbidities				
≥1, n (%)	0	11 (18.6 %)	39 (51.3 %)	<0.001
28-day mortality, n (%)	0	10 (16.9 %)	27 (35.5 %)	0.016

Data are shown as median and interquartile range unless otherwise indicated. Kruskal-Wallis one-way analysis of variance was performed for multi-group comparisons

*CNSI* central nervous system infection, *IAI* intra-abdominal infection, *MFI* mean of fluorescence intensities, *PD-1* Programmed cell death receptor-1, *PD-L1* programmed cell death receptor ligand-1, *SAPS II* simplified acute physiology score II, *SOFA* sepsis-related organ failure assessment, *USI* urinary system infection, *WBC* white blood cells

**Table 2:** Baseline characteristics of the patients with septic shock

Parameters	Survivors	Nonsurvivors	Overall population	*P* value
Number	49	27	76	–
Age (years)	73 (62–78)	70 (61–78)	71 (61–78)	0.765
Male, n (%)	22 (44.9 %)	15 (55.6 %)	37 (48.7 %)	0.792
WBC (×10^9^/L)	14.9 (12.4–16.9)	13.3 (10.9–17.8)	14.9 (11.8–17.2)	0.641
Lymphocyte (×10^9^/L)	0.78 (0.59–1.24)	0.65 (0.52–0.82)	0.76 (0.54–1.07)	0.046
SOFA score	11 (9–12)	14 (12–17)	11 (9–14)	<0.001
SAPS II	50 (43–56)	60 (53–66)	53 (46–60)	<0.001
Percentage of PD-1^+^/CD4^+^ T cells (%)	35.1 (28.5–44.8)	42.9 (34.9–49.0)	38.2 (29.2–47.7)	0.034
MFI of PD-1 on CD4^+^ T cells	7.0 (5.7–8.9)	8.8 (7.1–9.6)	7.5 (6.0–9.4)	0.036
Percentage of PD-1^+^/CD8^+^ T cells (%)	33.0 (24.8–44.3)	47.1 (30.3–53.9)	36.5 (27.3–51.3)	0.032
MFI of PD-1 on CD8^+^ T cells	5.9 (4.7–8.1)	7.8 (5.4–9.8)	6.6 (4.9–8.4)	0.033
Percentage of PD-L1^+^/CD4^+^ T cells (%)	16.4 (8.6–28.4)	22.1 (12.0–34.0)	21.0 (9.3–30.6)	0.295
MFI of PD-L1 on CD4^+^ T cells	1.9 (1.5–2.0)	1.8 (1.6–2.2)	1.8 (1.5–2.0)	0.961
Percentage of PD-L1^+^/CD8^+^ T cells (%)	23.2 (10.8–38.9)	12.9 (9.9–32.5)	19.5 (10.3–37.4)	0.204
MFI of PD-L1 on CD8^+^ T cells	1.6 (1.4–1.9)	1.5 (1.4–1.7)	1.5 (1.4–1.8)	0.378
Percentage of monocytes expressing PD-L1 (%)	28.9 (17.9–43.6)	53.9 (31.8–72.9)	35.9 (20.4–54.7)	0.001
MFI of PD-L1 on monocytes	8.2 (7.5–9.0)	9.1 (8.1–11.3)	8.3 (7.7–9.7)	0.012
Type of infection, n (%)				
Pneumonia	27 (55.1 %)	14 (51.9 %)	41 (53.9 %)	0.786
IAI	8 (16.3 %)	3 (11.1 %)	11 (14.5 %)	0.781
CNSI	4 (8.2 %)	2 (7.4 %)	6 (7.9 %)	0.743
USI	10 (20.4 %)	8 (29.6 %)	18 (23.7 %)	0.366
Number of comorbidities				
≥1, n (%)	21 (42.9 %)	18 (66.7 %)	39 (51.3 %)	0.047

Data are shown as median and interquartile range unless otherwise indicated

*CNSI* central nervous system infection, *IAI* intra-abdominal infection, *MFI* mean of fluorescence intensities, *PD-1* Programmed cell death receptor-1, *PD-L1* programmed cell death receptor ligand-1, *SAPS II* simplified acute physiology score II, *SOFA* sepsis-related organ failure assessment, *USI* urinary system infection, *WBC* white blood cells

### Comparison of median levels of PD-1 and PD-L1 expression

PD-1 and PD-L1 expression was measured on circulating CD4^+^ T cells, CD8^+^ T cells, and monocytes (PD-L1 only) at days 3–4 after the onset of SIRS (Table 1). Compared with healthy controls, the percentages of circulating PD-1/CD4^+^ T cells, PD-1/CD8^+^ T cells, and monocytes expressing PD-L1 were significantly higher in septic patients and septic shock patients (*P* < 0.05). Interestingly, only the percentages of monocytes expressing PD-L1 were obviously different between septic patients and septic shock patients (*P* < 0.05). Similar results were also observed when expressed as MFI.

### Correlation of PD-L1 expression with SOFA score or SAPS II in all patients

Spearman correlation analysis showed that the percentage of monocytes expressing PD-L1 in septic patients was positively correlated with SAPS II (*r* = 0.638; *P* < 0.001) and SOFA score (*r* = 0.654; *P* < 0.001). MFI of PD-L1 on monocytes was also positively correlated with SAPS II (*r* = 0.653; *P* < 0.001) and SOFA score (*r* = 0.582; *P* < 0.001).

### PD-1 and PD-L1 expression levels in survivors and nonsurvivors in septic shock patients

**Fig. 2:**
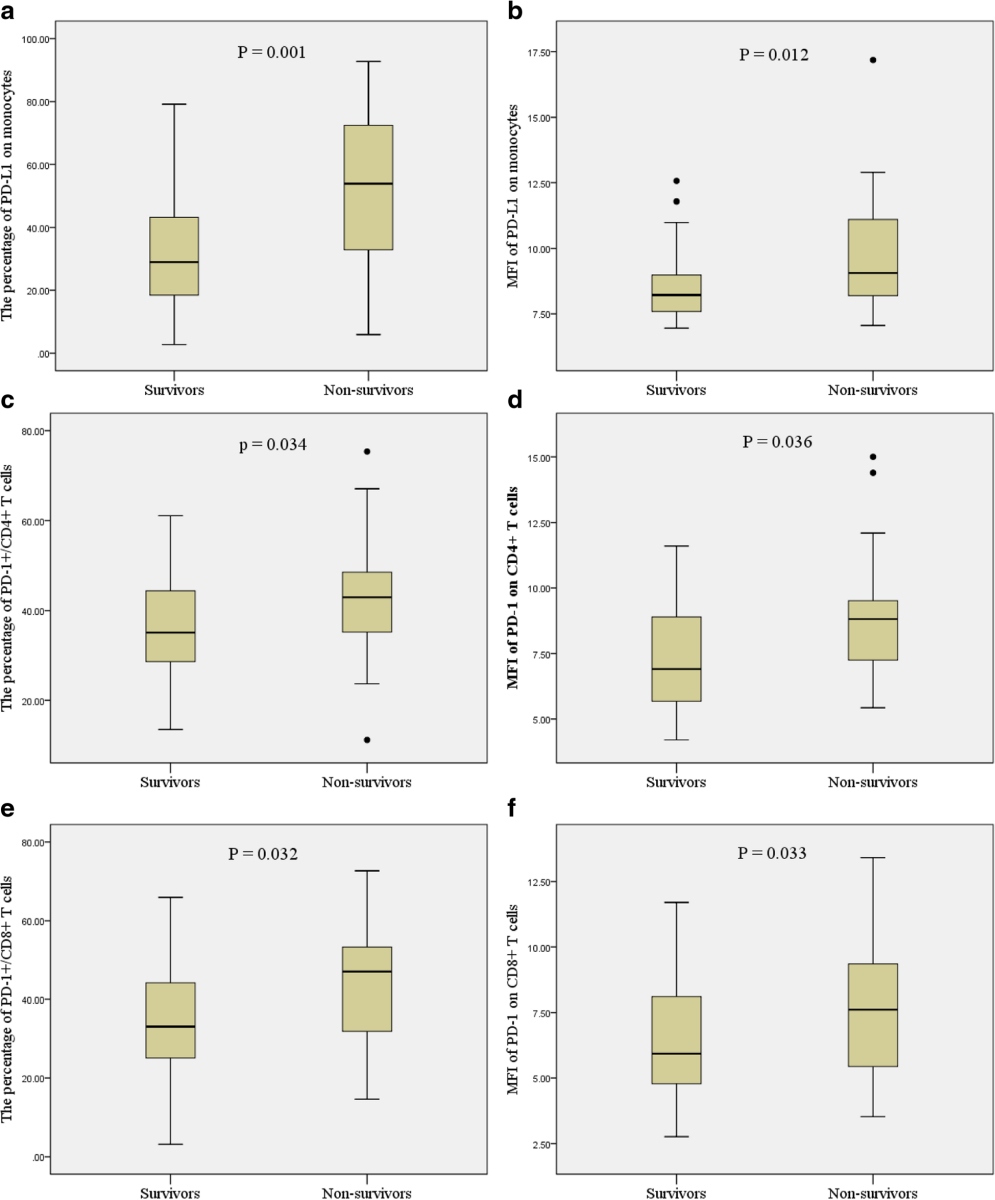
Box-plot representation of programmed cell death receptor-1 (*PD-1*)-related molecules. Data are shown as box plot with medians (*lines* inside boxes), 25th and 75th quartiles (limits of boxes); *whiskers* indicate the range. Programmed cell death receptor ligand-1 (*PD-L1*) expression on monocytes and PD-1 expression on T cells (CD4^+^ and CD8^+^ T cells) were significantly increased in nonsurvivors (*n* = 27) in comparison with survivors (*n* = 49) (**a–f**). *MFI* mean of fluorescence intensities

Septic shock patients were divided into survivors and nonsurvivors according to the 28-day mortality. In nonsurvivors, the percentages of circulating PD-1/CD4^+^ T cells, PD-1/CD8^+^ T cells, and monocytes expressing PD-L1 were significantly higher in comparison with survivors (Fig. 2; *P* < 0.05). Similar results were also observed when expressed as MFI (Fig. 2; *P* < 0.05).

### Value of PD-L1 expression for predicting 28-day mortality in septic shock patients

**Fig. 3:**
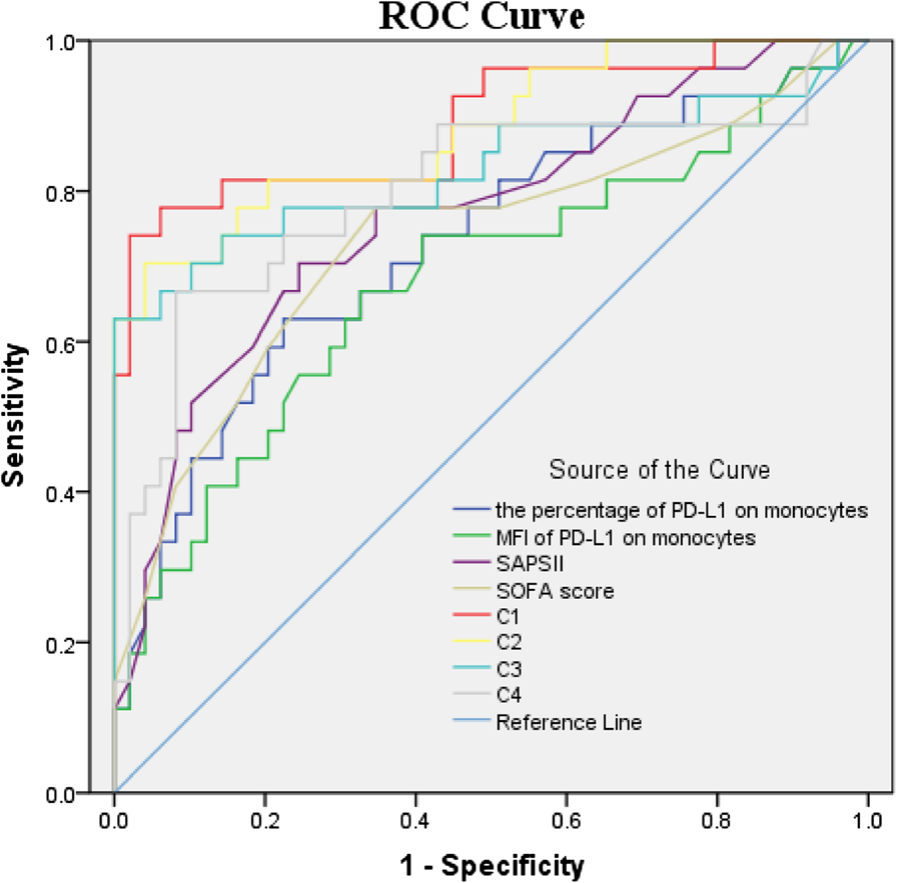
Receive operating characteristic (*ROC*) curve for predicting 28-day mortality in septic shock patients. AUCs: the percentage of PD-L1 on monocytes (*dark blue line*), 0.729; MFI of PD-L1 on monocytes (*green line*), 0.681; SAPS II (*pink line*), 0.768; SOFA score (*brown line*), 0.736; the percentage of PD-L1 on monocytes in combination with SAPS II (C1, *red line*), 0.891; MFI of PD-L1 on monocytes in combination with SAPS II (C2, *yellow line*), 0.881; the percentage of PD-L1 on monocytes in combination with SOFA score (C3, *pale blue line*), 0.829; and MFI of PD-L1 on monocytes in combination with SOFA score (C4, *gray line*), 0.799. *MFI* mean of fluorescence intensities, *PD-L1* programmed cell death receptor ligand-1, *SAPS II* simplified acute physiology score II, *SOFA* sepsis-related organ failure assessment

**Fig. 4:**
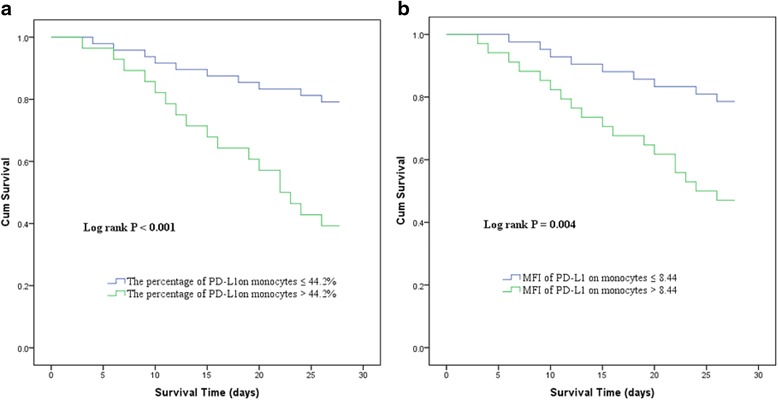
Survival curves of patients with septic shock according to the percentage of monocytes expressing PD-L1 (**a**) and MFI of PD-L1 on monocytes (**b**). *MFI* mean of fluorescence intensities, *PD-L1* programmed cell death receptor ligand-1

The ROC curve analysis (area under the curve (AUC)) showed that the percentage of monocytes expressing PD-L1 for predicting 28-day mortality was 0.729 (Table 3 and Fig. 3). ROC curve analysis showed that 44.2 % of monocytes expressing PD-L1 was the optimal threshold for predicting 28-day mortality in patients with septic shock. Using cutoff values determined by ROC, patients with a percentage of monocytes expressing PD-L1 higher than 44.2 % had a lower probability of survival at day 28 than patients with lower PD-L1 levels (Fig. 4; *P* < 0.001).

**Table 3:** Area under the curve of various parameters for predicting 28-day mortality in patients with septic shock

Variable	AUC	*P* value	95 % Confidence interval	
			Lower limit	Upper limit
Percentage of monocytes expressing PD-L1	0.729	0.001	0.607	0.852
MFI of PD-L1 on monocytes	0.681	0.009	0.548	0.813
SAPS II	0.768	<0.001	0.653	0.883
SOFA score	0.736	0.001	0.610	0.863
Percentage of PD-L1 on monocytes + SAPS II	0.891	<0.001	0.807	0.976
MFI of PD-L1 on monocytes + SAPS II	0.881	<0.001	0.797	0.965
Percentage of PD-L1 on monocytes + SOFA score	0.829	<0.001	0.713	0.944
MFI of PD-L1 on monocytes + SOFA score	0.799	<0.001	0.682	0.917

*AUC* area under the curve, *MFI* mean of fluorescence intensities, *PD-L1* programmed cell death receptor ligand-1, *SAPS II* simplified acute physiology score II, *SOFA* sepsis-related organ failure assessment

Using a cutoff value of 44.2 % (for the percentage of monocytes expressing PD-L1) for predicting 28-day mortality in patients with septic shock, the sensitivity was 68.0 %, specificity was 77.6 %, the positive predictive value (PPV) was 65.7 %, and the negative predictive value (NPV) was 79.2 %. Using a cutoff value of 8.44 (for MFI of PD-L1 on monocytes) for predicting 28-day mortality in patients with septic shock, the sensitivity was 66.7 %, specificity was 67.3 %, the PPV was 62.9 %, and the NPV was 78.6 %.

### PD-L1 expression as an independent predictor of 28-day mortality in septic shock patients

Univariate and multivariate logistic regression were used to identify PD-1-related molecules associated with 28-day mortality for patients with septic shock. Multivariate logistic regression analysis showed that only PD-L1 expression on monocytes was independently associated with 28-day mortality. The detailed data are presented in Table 4.

**Table 4:** Logistic regression analysis of independent factors for 28-day mortality in patients with septic shock

Variable	B	SE	Wald	*P* value	Odds ratio	95 % confidence interval for EXP(B)	
						Lower limit	Upper limit
Percentage of monocytes expressing PD-L1	1.943	0.745	6.811	0.009	6.981	1.622	30.041
Lymphocytes	0.088	0.794	0.012	0.912	1.092	0.230	5.173
SOFA score	1.550	0.687	5.091	0.024	4.713	1.226	18.117
SAPS II	1.558	0.723	4.646	0.031	4.749	1.152	19.584
Number of comorbidities	1.887	0.726	6.761	0.009	6.598	1.591	27.352
Constant	−4.133	0.918	20.256	0.000	0.016		

*PD-L1* programmed cell death receptor ligand-1, *SAPS II* simplified acute physiology score II, *SOFA* sepsis-related organ failure assessment

### Combination of PD-L1 expression with SOFA score or SAPS II in septic shock patients

We further explored the prognostic significance of a combination of independent predictors and conventional clinical risk parameters in septic shock patients. Interestingly, a combination of monocyte PD-L1 expression enhanced the ability of the SOFA score or SAPS II to predict 28-day mortality in patients with septic shock. The prognostic value of PD-L1 expression on monocytes in combination with SAPS II for predicting 28-day mortality was significantly higher than each parameter alone. The detailed data are presented in Table 3 and Fig. 3.

## Discussion

The present study demonstrated that, among PD-1-related molecules, only monocyte PD-L1 expression after 3–4 days of sepsis was valuable for the risk stratification of septic patients. Monocyte PD-L1 expression was also an independent predictor of mortality in septic shock patients. Additionally, our study demonstrated that monocyte PD-L1 expression combined with clinical risk parameter (i.e., SAPS II) could enhance the ability to predict 28-day mortality in patients with septic shock. To the best of our knowledge, this is the largest number of patients with sepsis for which PD-1-related molecules were explored, and our findings may be useful for septic patient stratification and prognosis evaluation.

Sepsis is a complex pathophysiological process. It is widely accepted that although there is a predominance of the hyperinflammatory phase after sepsis initiation, sepsis then rapidly develops a state of immunosuppression [17, 18]. Because of the application of antibiotics and other aggressive treatments, many patients may survive the initial proinflammatory stage, but eventually die later in a state of immunosuppression [18, 19]. PD-1 and its ligand PD-L1 are thought to play major roles in immunosuppressive mechanisms. Blockade of the PD-1/PD-L1 inhibitory pathway has been successfully used in cancer and septic animals, and may have similar beneficial effects in septic shock patients [20–23]. Since sepsis-induced immunosuppression may play an important role in mortality during sepsis, there is interest in identifying patients with sepsis who may benefit from anti-PD-1 or anti-PD-L1 antibody therapy. Our finding may be useful for subsequent studies of PD-1/PD-L1 blockade in sepsis.

Although previous studies showed that increased PD-1 expression on T cells and monocyte PD-L1 expression were associated with increased occurrence of mortality and nosocomial infections [24, 25], we observed that there were no differences in PD-1 expression on T cells between septic patients and septic shock patients, and also that it was not independently associated with 28-day mortality in patients with septic shock. Therefore, PD-1 expression on T cells may be not a reliable “danger signal” for immunosuppression in septic patients. Interestingly, we observed that monocyte PD-L1 expression was valuable for the risk stratification of septic patients, and that it was an independent predictor of mortality. In that sense, monocyte PD-L1 expression may be useful for patient stratification in clinical trials attempting to boost the immune system in septic patients. Patients with high levels of monocytes expressing PD-L1 should be considered as immunosuppressed, which should be taken into consideration regarding potentially deleterious effects.

Previous studies have demonstrated that anti-PD-1 and anti-PD-L1 antibodies could inhibit lymphocyte apoptosis, reverse immune dysfunction, attenuate organ dysfunction, and improve survival in a murine model of sepsis [21, 25, 26]. In that sense, PD-1 and PD-L1 were ideal targets to restore immune status in sepsis. The reasons may be due to the fact that PD-L1 plays a major role in the PD-1/PD-L1 pathway by exerting inhibitory effects, while PD-1 is regarded as an auxiliary part of that process. Previous mechanistic studies of PD-L1 provided several lines of evidence in support of this point: PD-L1 blockage altered plasma cytokine levels, but PD-1 blockage did not [20, 25]. Our study showed that PD-L1 expression on monocytes was independently associated with 28-day mortality in patients with septic shock, but PD-1 on T cells was not; more studies are still needed to certify this conclusion.

Another intriguing finding of our study was that a combination of monocyte PD-L1 expression with SAPS II or SOFA score significantly enhanced the accuracy of predicting 28-day mortality in patients with septic shock. The optimized information provided by the use of combined markers was clearly illustrated in our study. Conventional clinical risk parameters indicated the severity of disease. The levels of co-inhibitory receptor molecules indicated the degree of immunosuppression that is characteristic of an inefficient clearance of invasive microbial pathogens. Combination of monocyte PD-L1 expression and conventional clinical risk parameters may be optimal candidates for predicting the prognosis of septic shock. Of particular importance, we demonstrated that monocyte PD-L1 expression combined with SAPS II was the best factor for predicting 28-day mortality in septic shock patients. These results suggested that monocyte PD-L1 expression may enhance the ability of clinical markers to evaluate the prognosis of septic shock patients.

Some limitations need to be considered in our study. First, the sample size was relatively small, and it was a single-center study. The current study findings should be confirmed by a multicenter study. Second, dynamic changes in the levels of monocyte PD-L1 expression remain targets for future studies. Third, because of the differences in the brand of flow cytometer used in different institutions, the cutoff values of PD-L1 on monocytes to predict poor outcome may be different. Therefore, similar to monocyte HLA-DR measurement [27], it is necessary to establish a standard protocol in different institutions. Previous studies have demonstrated that low levels of monocyte HLA-DR expression were associated with impairment of monocytic cellular functions [28, 29]. Correlation of PD-L1 values with HLA-DR expression may provide more useful information.

## Conclusions

In conclusion, among PD-1-related molecules only monocyte PD-L1 expression after 3–4 days of sepsis is associated with risk stratification and mortality in septic patients. Furthermore, measurement of monocyte PD-L1 expression was a promising independent prognostic marker for septic shock patients.

## Abbreviations

AUC: area under the curve
ED: emergency department
EDTA: ethylenediamine tetraacetic acid
FSC: forward scatter
HLA-DR: human leukocyte antigen-DR
MAP: mean arterial pressure
MFI: mean of fluorescence intensities
NPV: negative predictive value
PD-1: programmed cell death receptor-1
PD-L1: programmed cell death receptor ligand-1
PPV: positive predictive value
ROC: receiver operating characteristic curve
SAPS II: simplified acute physiology score II
SBP: systolic blood pressure
SIRS: system inflammatory response syndrome
SOFA: Sepsis-related organ failure assessment
SSC: side scatter
USI: urinary system infection
WBC: white blood cell
